# A Pilot Study to Assess the Feasibility of the Spanish Diabetes Self-Management Program in the Basque Country

**DOI:** 10.1155/2016/9145673

**Published:** 2016-12-29

**Authors:** Estibaliz Gamboa Moreno, Lourdes Ochoa de Retana Garcia, Maria Emma del Campo Pena, Álvaro Sánchez Perez, Catalina Martinez Carazo, Juan Carlos Arbonies Ortiz, Maria Angeles Rua Portu, Koldo Piñera Elorriaga, Amaya Zenarutzabeitia Pikatza, Miren Nekane Urquiza Bengoa, Tomás Méndez Sanpedro, Ana Oses Portu, Lourdes Gorostidi Fano, Miren Bakarne Aguirre Sorondo, Kalliopi Vrotsou, Rafael Rotaeche Del Campo

**Affiliations:** ^1^Active Patient Program, Donostialdea Integrated Health Organisation, Osakidetza, Pasajes San Pedro Health Center, Guipuzcoa, Spain; ^2^Donostialdea Integrated Health Organization, Osakidetza, Pasajes San Pedro Health Center, C/Marinos No. 1, Pasajes, San Pedro, 20110 Guipuzcoa, Spain; ^3^Research Unit, Primary Care-Organization of Integrated Health Services of Vizcaya, Osakidetza, Bilbao, Spain; ^4^Donostialdea Integrated Health Organization, Osakidetza, Beraun Health Center, Renteria, Guipuzcoa, Spain; ^5^Donostialdea Integrated Health Organization, Osakidetza, Bidebieta Health Center, San Sebastián, Spain; ^6^O + Berri, Basque Institute for Healthcare Innovation, Barakaldo, Vizcaya, Spain; ^7^Family Medicine and Community Teaching Unit of Vizcaya, Osakidetza, Bilbao, Spain; ^8^Araba Area, Osakidetza, Olaguibel Health Center, Vitoria-Gasteiz, Spain; ^9^Ezkerraldea Enkarterri Cruces Integrated Health Organization, Osakidetza, Ortuella Health Center, Ortuella, Vizcaya, Spain; ^10^Bidasoa Integrated Health Organization, Osakidetza, Hondarribia Health Center, Hondarribia, Guipuzcoa, Spain; ^11^Research Unit, Primary Care-Organization of Integrated Health Services of Guipuzcoa, Osakidetza, San Sebastián, Spain; ^12^Donostialdea Integrated Health Organization, Osakidetza, Alza Health Center, San Sebastián, Spain

## Abstract

*Purpose*. The purpose of this study was to assess the feasibility of the Spanish Diabetes Self-Management Program (SDSMP) in the primary care setting of the Basque Health Service and offer initial estimations of the randomized controlled trial (RCT) effects.* Methods*. Ten health centers (HCs) participated in a single-arm pilot study with a 6-month follow-up period between February 2011 and June 2012. Recruitment was performed via invitation letters, health professionals, and the local media. Each intervention group consisted of 8–15 people. The ability of each HC in forming up to 2 groups, participants' compliance with the course, and coordination and data collection issues were evaluated. Glycated haemoglobin (HbA1c) was the main outcome variable. Secondary outcomes were cardiovascular risk factors, drugs consumption, medical visits, quality of life, self-efficacy, physical exercise, and diet.* Results*. Two HCs did not organize a course. A total of 173 patients initiated the program, 2 dropped out without baseline data, and 90% completed it. No pre-post HbA1c differences existed. Certain improvements were observed in blood pressure control, self-efficacy, physical activity, and some dietary habits.* Conclusion*. The SDSMP is feasible in our setting. Our experience can be of interest when planning and conducting this program in similar health settings. The trial is registered with ClinicalTrials.gov identifier NCT01642394.

## 1. Introduction

Diabetes is one of the most prevalent chronic diseases, with 422 million people worldwide having diagnosed diabetes in 2014 [1]. It is estimated that the prevalence of diagnosed and undiagnosed type 2 diabetes (T2DM) in Spain may reach as high as 12% in people above 30 years of age [2]. T2DM is associated with an increased morbidity and mortality and it is thought to be responsible for 1.5 million deaths in 2012 [3]. What is more, the direct and indirect costs of the disease between 2011 and 2030 will reach US $ 1.7 trillion [3]. It is estimated that diabetes accounts for between 6.3 and 7.4% of the costs in our health system [4].

Above all, T2DM is associated with cardiovascular system conditions, such problems being the cause of death in three-quarters of the patients. In the Basque Country, 44% of people with diabetes are obese [4] and 22% have diabetic macroangiopathy [5]. The diabetes control is improving in our setting, although 64% of patients have HbA1c levels below 7% and only 50% have blood pressure readings under 140/80 mmHg [5].

Patients' education can play an important role in improving glycemic control and reducing cardiovascular risk [6]. The current Basque Country clinical practice guideline (GPC) for T2DM recommends offering a structured educational program in order to empower the patients and encourage their active participation in the management of their condition [6]. Patient activation is defined as understanding one's own role in the care process and having the knowledge, skills, and confidence to take on that role [7].

Research indicates that activated patients are more likely to adhere to treatment regimens, get preventive care, and participate to a greater degree in decisions about their care [8]. They are also more likely to engage in healthy lifestyle behaviors, to seek out health information, and to make less use of healthcare services. Interventions that provide peer support for patients and improve their problem-solving skills have also been shown to increase patient activation and improve health outcomes [8].

Like other chronic illnesses, diabetes requires patients to take responsibility for their own health (self-care) to minimize long-term complications. Among programs on patient self- management, the most widely used structured approach is the Chronic Disease Self-Management Program (CDSMP) [9] developed at Stanford University. The CDSMP has different versions, among which is the Diabetes Self-Management Program (DSMP), specifically adapted for T2DM patients and its Spanish language version (SDSMP) [10]. These self-management programs are based on Albert Bandura's self-efficacy theory of behavioral change [11], which states that the key predictive variables for successful change are confidence (self-efficacy) in the capacity for carrying out an action and the expectation of achieving a particular goal (outcome expectation). Many studies support that self-efficacy and changes seen in the latter are associated with changes in health behavior and health status [12].

Successive systematic reviews have been published on the efficacy of various educational models in self-care and patient activation [13–16]. These reviews indicate great variety in the results of the interventions attributable to differences in the length of the follow-up, the modality of the interventions, and the target populations.

In Spain, the few data available on self-management programs suggest favorable results, but the latter have not been assessed through prospective studies or compared with usual care [17]. The Department of Health of the Basque Country has launched a new strategy for providing care to chronic patients based on the Chronic Care Model [18]. One of the cornerstones of this model is the promotion of self-care and population education. In this context, one of the trainings that offers the Active Patient Program (“*Paciente Activo*”) follows the SDSMP methodology and has been proposed as an instrument to promote self-care in people with T2DM. In our health system, these educational interventions take place in the primary care health centers. These centers mainly host general practitioners, pediatricians, and nurses, with the latter bearing the responsibility of most educational activities.

A single-arm pilot study was conducted for assessing the feasibility of this educational intervention in our context [19]. Acceptability, participation, and satisfaction with the educational intervention were studied. Furthermore, the obtained data served to estimate the subsequent clinical trial sample size [19], while offering initial estimations of the expected effects for the main and secondary outcomes [19]. Finally, the experiences and lessons learned during this phase helped the investigators to better prepare, organize, and control all aspects of the subsequent clinical trial [20].

## 2. Objectives

The feasibility aspects assessed by the pilot study were the interest of the target population in the proposed educational program and the enrollment rate; compliance with the program's schedule; adequacy of the battery of the administered questions, and finally participation and coordination of several health centers (HCs).

In addition, the main and secondary outcome pre-post effects were estimated. The standard deviation (SD) estimation of the main outcome of interest (HbA1c) was implemented in the sample size estimation of the clinical trial. All primary and secondary derived effects offer an initial idea of the results that may be expected in the clinical trial.

## 3. Methods

This preliminary research was a prospective pre-post pilot study without a control group. Recruitment took place in 10 participating HCs across 4 healthcare organizations (i.e., primary care districts of Araba, Gipuzkoa, Ezkerraldea-Enkarterri, and Bidasoa Integrated Healthcare Organization) in the Basque Country (Spain) from February 2011 to June 2012. Between 2 and 9 health professionals (HPs) participated per center.

Patients with T2DM between the ages of 18 and 79 years were included. Individuals with mental health problems (bipolar disorder, psychosis, schizophrenia, Alzheimer's disease, or other forms of dementia) or other health problems, that might have affected their ability to participate in the study, were excluded.

Recruitment was carried out in several ways. Invitations letters were sent to 120 T2DM subjects of each participating HC, fulfilling the age criteria. These subjects were selected via a computer generated random numbers sequence. In addition, the participating HPs were instructed to inform and invite patients to the study. Finally, awareness about the program was also spread in the local media. All the patients who agreed to participate gave written informed consent, after receiving information about the purpose of the research project.

Sociodemographic and baseline clinical data were collected on age, sex, years since diagnosis, and comorbidities. Assessed comorbidities were hypertension, heart disease, macroangiopathy (coronary, cerebrovascular, or peripheral artery disease), microangiopathy (renal, retinopathy, or neuropathy), depression, asthma, chronic obstructive pulmonary disease, and cancer.

## 4. Description of the Intervention

Self-efficacy enhancement was the key element of the applied educational intervention. The teaching process was structured to include the following four self-efficacy components: performance mastery, which shows participants how to make specific action plans; modeling, which can be accomplished by involving peers as instructors of self-management programs; symptom interpretation, helping patients to form alternative interpretations of their physiological symptoms, as such interpretations can subsequently lead to new self-management behaviors; and, finally, social persuasion, which refers to the positive effect experienced by the majority of the group members and the way in which this can influence other group members [12]. On the other hand, the content of the self-management program addressed three tasks, medical or behavioral management, role management, and emotional management, and five core skills, problem solving, decision-making, resource utilization, forming a patient/healthcare provider partnership, and taking action [21]. The intervention consisted of 6 group sessions lasting 2.5 hours each, once a week for 6 weeks. Sessions were structured with the objective of enabling participants to acquire knowledge and skills related to the disease and its management, placing emphasis on tools for enhancing proactive self-care to achieve healthier lifestyle behaviors (improvements in diet, physical activity patterns, emotional management, and medication adherence among others).

Patients were trained to set their own targets, solve problems related to their condition, and communicate more effectively, with their relatives and healthcare professionals, by sharing their feelings, in order to enable them to play a more active role in the management of their disease. The final goal of all this was to promote changes towards healthier lifestyles.

All sessions were supported by educational material specifically developed for the program: books, leaflets, and CDs. Each group was supervised by two leaders previously trained and certified in the SDSMP. At least one of the leaders was required to be a T2DM patient or a caregiver for a person with this condition, while the other was allowed to be a HP. These leaders introduced themselves to participants as SDSMP leaders, not referring to their professional position, promoting the concept of peer-learning, as recommended in the implementation manual of the SDSMP. Patients not attending at least four sessions were considered not to have completed the program.

## 5. Outcomes

### 5.1. Feasibility Assessment

The recruitment capacity of the centers and their ability in forming up to 2 intervention groups each was assessed. Each center was asked to recruit between 8 and 30 subjects. For the needs of this study, 8 and 15 were the minimum and maximum acceptable number of participants in any group. At least 65% of the participants initiating the intervention were expected to complete it [15]. Each center was responsible for and should be successful at managing all program related aspects and data collection. The actual educational intervention was delivered by the same investigators across all centers. Finally, the principal investigators attended interested patients, corresponding to nonparticipating HCs, organized the details related to baseline data information, and referred those subjects to the most convenient participating HC for receiving the educational intervention. The adequacy and understanding of the battery of questions would be judged by the frequency of missing data, while at the same time this would also inform about appropriate patient follow-up. A manual with detailed instructions related to the pilot study project was given to all participating HPs.

### 5.2. Clinical Outcomes

#### 5.2.1. Primary Outcome Variable

 Glycated haemoglobin (HbA1c) level was a primary outcome variable.

#### 5.2.2. Secondary Outcome Variables

*Cardiovascular-Related Factors.* The factors are body mass index (BMI), systolic and diastolic blood pressure (SBP and DBP), and total and HDL cholesterol levels. Cardiovascular risk was assessed with the* Registre Gironí del COR *(REGICOR) score, an adaptation of the original Framingham risk score for Mediterranean populations, calculated for persons between 35 and 74 years of age [22].

*Use of Medications.* Antidiabetes, antihypertensives, and antiplatelets were studied. 

*Quality of Life. *The Spanish version of the self-administered instrument, Audit of Diabetes-Dependent Quality of Life (ADDQoL-19), was used [23]. This scale is specific for patients with diabetes and consists of 19 items assessing leisure activities, relationships, and living conditions. All items are addressed from two perspectives: the way diabetes affects the patient's life and what a patient's life might be like if they did not have diabetes. Replies range from 1 (excellent) to 7 (very poor).

*Self-Efficacy.* The Spanish Diabetes Self-Efficacy Scale developed at Stanford University [24] was administered. It consists of 8 items assessing diet, physical activity, and control of the disease. Items are rated on a Likert-type scale from 1 to 10 (minimum to maximum). A total score and scores for the three aforementioned areas were obtained.

*Physical Exercise.* Physical exercise was assessed with the 7-Day Physical Activity Recall (PAR) interview [25, 26]. This is a semistructured interview concerning the intensity of physical activity performed in the previous week. Exercise intensity in metabolic equivalents (METs) in hours/week is estimated considering the hours of moderate, intense, and very intense exercise. PAR also assesses whether the exercise reported by the patients is suitable for their age.

*Diet. *Diet quality was examined using the food frequency questionnaire of the PREDIMED study [27]. This questionnaire assesses frequency consumption of olive oil, fruit, vegetables, dairy products, cereal, red and white meat, fish, pasta or rice, legumes, commercial sweets, and beverages.

*Patient Satisfaction with the Program. *It was measured with an anonymous specific satisfaction survey consisting of 10 questions related to sociodemographic and process variables, 20 satisfaction questions rated on 5-point Likert-type scale ranging from 1 = minimum to 5 = maximum and 3 open questions. Questions were divided into three sections referring to the material presented, organization of the program, and behavior change (see Supplementary Material available online at http://dx.doi.org/10.1155/2016/9145673).

*Use of Healthcare Services. *Number of visits to the general practitioner and nurse, number of visits to the emergency department, and number of times of hospitalization are compared during a 6-month period before and after the intervention. Only cardiovascular morbidity and diabetes-related complications (e.g., renal insufficiency, hypoglycemia, and ketoacidosis) were considered for the emergency department visits and hospital admissions.

Patients were assessed twice, 1 month before starting the intervention and 6 months after the end of the intervention. Sociodemographic and self-report questionnaires were given to patients to fill in, in their own homes. All participating HPs, previously trained by the research team, were in charge of collecting the following data. Medication consumption and clinical visits were assessed from the electronic clinical history files and corroborated by the participants. Body mass index (BMI) and systolic (SBP) and diastolic blood pressure (DBP) were recorded, while HbA1c and cholesterol levels were assessed by blood samples. These samples were extracted in participants' HCs and were analyzed in the reference laboratories of the four participating health districts of the Basque Health System (Osakidetza). When necessary, the referring HP helped the participants complete the questionnaires.

Finally, the 7-Day PAR and PREDIMED questionnaires were administered over the telephone by trained interviewers from a centralized call center.

## 6. Statistical Analysis

Categorical variables were expressed as frequencies (*n*) and percentages (%) and continuous variables as means and standard deviations (SD) when normally distributed or as medians and interquartile ranges (Q1, Q3) when they did not follow a normal distribution.

The comparisons between categorical variables before and after the intervention were carried out with McNemar's test. Comparisons between continuous variables were performed with Student's* t* test for paired samples or the nonparametric Wilcoxon signed-rank test. All the differences were calculated as postintervention minus preintervention values. For normally distributed variables, differences are presented as means with 95% confidence intervals (CI), while, for nonnormally distributed variables, such as the number of visits, differences are expressed as medians and their corresponding 95% CI. Comparisons were considered as statistically significant when *p* < 0.05. For the needs of this study, all results are based on available data. Statistical analyses were carried out using the SAS v.9.3.

## 7. Ethical Considerations

The research protocol was approved by the Clinical Research Ethics Committee of the Basque Country (Ref. number: 11/2010).

## 8. Results

### 8.1. Feasibility Assessment

The participating HCs recruited between 3 and 27 patients each, while 5 patients corresponded to nonparticipating HCs. Five of the centers obtained two program groups; three centers obtained one group, while patients recruited in two centers (i.e., *n* = 3 and 5) had to follow the program in a different HC, for being less than the required minimum for a course. The 5 additional subjects were absorbed without affecting the respective number of program groups. Of the 1200 invitation letters sent, 46 were undelivered. Many patients visited their corresponding HP with an invitation letter and requested more details on the program. However, frequency of patients who responded to the letter's invitation, of patients who showed initial interest, and of patients informed exclusively by the HP was not registered.

**Figure 1:**
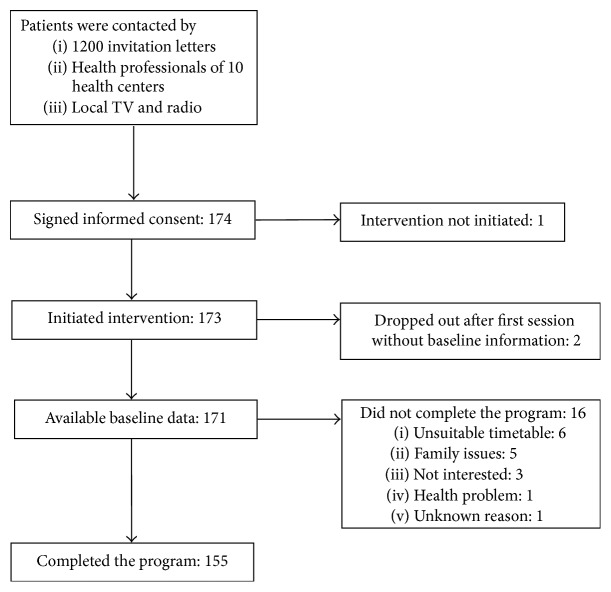
Flowchart of pilot study participants.

A total of 174 patients signed an informed consent, 173 initiated the program, and 2 dropped out after the first session, without providing baseline data. One hundred and fifty-five patients (90%) completed the training program (Figure 1).

Between 1 and 9% of the self-reported questions were not answered at baseline. At the postintervention assessment, the main outcome of interest, along with other cardiovascular data, was missing for 5 of the 171 participants, while the amount of missing data of the self-reported variables had increased.

### 8.2. Baseline Characteristics of Participants

Overall, 52% of the sample were male, with mean (SD) age of 63.4 (8.1) years. Among the most common comorbidities were hypertension (57%) and heart disease (26%), while 25% and 14% of the participants had a history of macro- and microangiopathy, respectively. Baseline data are summarized in Table 1.

### 8.3. Pre-Post Differences

HbA1c levels at the two pilot study moments were 7.3% (1.1) and 7.4% (1.3), respectively, with the mean difference between the measurements being 0.1% (95% CI: −0.1 to 0.2; *p* = 0.348) (Table 2). It was additionally assessed whether patients with poorer initial control, defined as a baseline HbA1c ≥ 7%, presented greater reductions in this variable, but no differences were observed neither in patients with HbA1c ≥ 7% (diff: 0.01 (95% CI: −0.21 to 0.22); *p* = 0.960; *n* = 94) nor in patients with HbA1c ≥ 8% (diff: −0.06 (95% CI: −0.54 to 0.42); *p* = 0.791; *n* = 38) between the two time points. Further, no differences in BMI, total cholesterol levels, or cardiovascular risk were found (Table 2).

In terms of blood pressure, a reduction was seen after the intervention. The mean changes in SBP and DBP were −3.3 mmHg (95% CI: −5.4 to −1.3; *p* = 0.002) and −1.3 mmHg (95% CI: −2.5 to −0.1; *p* = 0.032), respectively. These reductions were also reflected in an increased percentage of patients who simultaneously achieved good control of both SBP and DBP (SBP < 140 and DBP < 90 mmHg) after the intervention, with 10% (95% CI: 3 to 18; *p* = 0.010) of the participants improving the control of their blood pressure during the study. However, this improvement was not reflected in a coronary risk reduction (Table 2).

In addition, the pilot study participants reduced by 1 both general practitioner (*p* = 0.005) and primary nurse visits (*p* < 0.0001). Frequency of emergency department visits and hospitalization remained 0 at both time points. Finally, no differences were seen neither in the total number of medications per patient nor in the percentage of patients taking antidiabetics, antihypertensives, or antiplatelets drugs (Table 2).

When replying to the general item of the ADDQoL-19 “In general, my present quality of life is…” participants rated their quality of life as being better 6 months after the pilot study (*p* = 0.027). On the other hand, no differences were observed in the general item, “If I did not have diabetes, my quality of life would be…” (*p* = 0.263) or in the total ADDQoL-19 score (*p* = 0.877) between the two moments (Table 3).

Self-efficacy significantly improved both overall and in the different areas, namely, diet, physical activity, and control of the disease. The observed changes ranged from 0.5 (95% CI: 0.1 to 0.9) to 0.8 (95% CI: 0.5 to 1.2) (Table 3).

The percentage of participants who reached the recommended levels of physical activity for their age increased 6 months after the intervention by 12% (95% CI: 4 to 21%; *p* = 0.007), while this improvement was not captured when physical activity was measured in minutes and METs (Table 3). Regarding dietary habits, a 10% increase was observed in the percentage of patients eating five or more portions of fruit and vegetables after the intervention (*p* = 0.020) and cold cured meats consumption was reduced (*p* = 0.035). However, none of the other main dietary habits was altered (Table 3).

As far as satisfaction with the course was concerned, a total of 149 patients replied to these questions. In 19 of the 20 questions, the median score was 5 (95% CI: 4-5), with only one item “This course is going to help me to manage my emotions better,” having a lower median score of 4 (95% CI: 4-5).

## 9. Discussion and Conclusion

### 9.1. Discussion

Based on the current pilot study, we concluded that performing a randomized clinical trial (RCT) for evaluating the effectiveness of an educational program for diabetic patients was feasible in our context. Results were acceptable as far as overall recruitment, course participation, and patients' satisfaction and collaboration across various centers was concerned.

However, several important observations were also made. Two of the participating centers, with 3 HPs collaborators each, did not manage to fulfill the minimum number of required participants. The number of involved staff, their motivation, and understanding of the study goals were important aspects to consider in the future RCT. This was seen as a key aspect for the successful RCT fulfillment, especially considering that the latter would involve a great number of centers dispersed over the whole Basque Country. Therefore, it was decided that at least 5 or 6 HPs per center should be achieved for the future study. In addition, during the RCT informative sessions for capturing participating HPs, more effort should be made on highlighting the positive aspects of the educational intervention; this was expected to improve patients' health control and relieve, in the long term, the workload of the professionals themselves. It was also thought that the HP motivation would increase, if pilot study patients participated actively in those informative sessions.

During the pilot study many participating HPs complained that their workload did not allow them to devote any time to the current project. For this reason, HPs participating in the RCT were going to be allowed (by Osakidetza) a certain amount of working hours devoted exclusively to the needs of that study.

It was also observed that missing information, especially on subjective and patient self-reported data, increased at six months postintervention, compared to baseline. This fact was taken into consideration when estimating the RCT sample size, but also it indicated the need for a closer patient follow-up during the RCT data collection.

The pre-post differences obtained in the current single-arm study may offer an initial estimation of the expected RCT results. The baseline characteristics of the current sample were comparable to those of the average diabetic patient in the Basque Country [4], except in that they were slightly younger and had lower levels of total cholesterol.

Changes in several dimensions including improvements in the self-efficacy scale, levels of exercise, and diet were observed. However, these changes were not accompanied by a greater glycaemic control in terms of HbA1c levels or changes in other variables, related to vascular morbidity, like coronary risk for example. The good initial control of the local T2DM population and the short follow-up of the pilot study could be possible explanations of this lack of difference. It is recognized that diabetic people with poorer HbA1c levels have a greater room for improvement and tend to respond better to any type of intervention [4]. However, this phenomenon has not been observed consistently in the context of the Stanford Self-Management Programs [28–31]. This very hypothesis will be tested in the RCT study, where a greater number of subjects will be followed for a longer period of time.

It is important to note that although the target HbA1c level is the most widely used variable to date for assessing diabetes interventions, its use as the only method is currently being questioned. Recent evidence has shown that lower HbA1c levels are not always accompanied by a decrease in cardiovascular morbidity and mortality, especially in older patients or those with comorbidities [32]. Hence, it may be necessary to adapt the selection of variables to assess diabetes control to baseline levels of HbA1c, the length of time since diagnosis of the disease, and the presence of risk factors and cardiovascular morbidity [33]. The following RCT study of this group will explore the HbA1c level as its main outcome, in order to confirm or refute the prior theories, after a 2-year follow-up period, in our context.

The pilot study results suggested a reduction in blood pressure, similar to that obtained using nondrug approaches, such as a salt-free diet and physical exercise, and this was reflected to a significant increase in the percentage of patients with good blood pressure control, without changing the prescription of antihypertensives. The effect on blood pressure has not been included in any of the evaluations of the DSMP we identified in our review of the literature [28, 34, 35]. Given the high prevalence of hypertension in people with diabetes and the importance of decreasing blood pressure for reducing cardiovascular morbidity and mortality, this potentially promising finding should be confirmed in future prospective studies, while the influence of a blood pressure reduction in the cardiovascular risk should also be further explored [20].

An improvement in scores on the specific self-efficacy questionnaire is a common finding in all evaluations on diabetes self-management [15, 36, 37]. However, in many occasions, the significant progress achieved was not followed by an improvement in quality of life [15]. On the other hand, people with diabetes, who improve their disease knowledge and self-management skills, are more independent and use fewer healthcare resources. This well-documented finding [15, 36, 37] was also observed in our sample.

The effects seen in the current study cannot be generalized and no causal relationship can be claimed between those effects and the applied educational intervention. The fact that the DSMP has been assessed in other populations and contexts, resulting in modest short-term positive results in outcomes such as depression, dietary habits, exercise, medication adherence, symptoms of hypoglycemia, communication with physicians, and health status [34–36], make us believe that the RCT findings will be in line with the a priori positive expectations.

This pilot study has certain limitations. Firstly, its single-arm nature was not a replicate of the future RCT study. However, its design permitted testing the feasibility of the educational program, in terms of participation and multiple-sites coordination. Even though the minimum number of participants was met overall, great variability was seen among participating HCs. Motivation and time availability of participating HPs are issues that will be treated more carefully in the follow-up RCT study. What is more, exact patient participation rates cannot be calculated. On one hand, the invitation letters were sent to T2DM patients, irrespective of whether or not they were fulfilling any other inclusion criteria than the age. This was due to the data confidentially, which did not permit access to further medical information of the population of interest. Furthermore, no records of interested patients and patients informed exclusively by the HPs were kept. However, given the number of the participating centers, health professional and recruitment approaches, such a record would have been very difficult to manage. Finally, missing data information, especially at 6 months, is another important limitation. Patients that were not satisfied may have been more reluctant to reply after the intervention and thus the initially obtained and presented results may have been biased. Given the importance of missing and the effect they can have on the study's conclusions, a closer patient follow-up, at all stages, should be assured in the RCT.

### 9.2. Conclusion

The Spanish Diabetes Self-Management Program is feasible in our health system and well accepted by patients. The single-arm pilot study results suggest that the program may induce improvements in self-efficacy and blood pressure, but its effectiveness will have to be confirmed by a RCT.

## 10. Practice Implications

The current pilot study could contribute to the debate on the most adequate outcome variables, for evaluating diabetic patients' interventions. In addition, the experiences and lessons learned during this phase will serve for better coordinating the future RCT study. Avoiding organizational and communicational flaws, motivating HPs, improving patients' follow-up, and refining the study manual will result in enhancing the RCT quality at all levels. Finally, the experience and lessons learned may be beneficial to similar health settings for planning and conducting diabetes self-management programs.

## Supplementary Material

Satisfaction survey questions.

## Figures and Tables

**Table 1:** Baseline characteristics of participating patients.

Baseline information	*N* = 171
Age in years; mean (SD)	63.4 (8.1)
Sex; *n* (%)	
Male	89 (52)
Female	82 (48)
Years with diabetes; mean (SD)	9.7 (7.2)
Smoking status; *n* (%)	
Smoker	30 (18)
Nonsmoker	141 (82)
Comorbidities; *n* (%)	
Hypertension	97 (57)
Heart disease	44 (26)
Macroangiopathy	43 (25)
Microangiopathy	24 (14)

*n *(%) = frequency (percentage) and SD = standard deviation. Comorbidity data indicate frequency of the “yes” category.

**Table 2:** Cardiovascular clinical variables and number of medical visits and times of hospitalization at baseline and 6 months after the intervention.

Variables	*n*	Preintervention	Mean difference (95% CI)	*p* value
*Cardiovascular data*				
HbA1c level	166	7.3 (1.1)	0.1 (−0.1, 0.2)	0.348
HbA1c < 7%; *n* (%)		72 (43)	3 (−4, 9)	0.465
BMI	167	30.4 (5.3)	−0.1 (−0.3, 0.1)	0.461
Total cholesterol	164	197.8 (37.9)	−3.2 (−7.6, 1.2)	0.158
REGICOR score	145	7.2 (3.8)	−0.2 (−0.6, 0.3)	0.466
SBP	166	137.1 (16.6)	−3.3 (−5.3, −1.2)	0.002
DBP	166	79.2 (9.9)	−1.4 (−2.5, −0.2)	0.024
Good blood pressure control SBP < 140 & DBP < 90; *n* (%)	166	88 (53)	10 (3, 18)	0.007
*Medication consumption*	171			
Antidiabetics; *n* (%)		133 (78)	1 (−2, 4)	0.479
Antihypertensives; *n* (%)		98 (57)	0 (−4, 4)	1.000
Antiplatelet drugs; *n* (%)		56 (33)	1 (−5, 2)	0.527
Number of medications; median (Q1, Q3)		3 (1, 4)	0 (0, 0)	0.763
*Number of medical visits; * *median (Q1,Q3)*	165			
General practitioner		3 (2, 5)	−1 (−1, 0)	0.005
Primary care nurse		4 (2, 5)	−1 (−1, −1)	<0.0001
Emergency department		0 (0, 0)	0 (0, 0)	0.815
Hospital admissions		0 (0, 0)	0 (0, 0)	0.278

Data are mean (standard deviation), unless otherwise stated. *n* (%) = frequency (percentage). The “*n*” column reports frequencies of available data at both time points. REGICOR estimates cardiovascular risk for patients between 35 and 74 years of age. Differences were calculated as postintervention minus preintervention values. CI: confidence interval. Q1, Q3: 25th and 75th percentiles. HbA1c: glycated haemoglobin. BMI: body mass index. SBP: systolic blood pressure; DBP: diastolic blood pressure. Mean difference for categorical variables corresponds to differences in paired proportions and their respective 95% CI and for ordinal variables (i.e., total number of medications and medical visits) to median differences with their respective 95% CI. Reported *p* values are based on paired *t*-tests for continuous variables, McNemar's test for binary variables, and the Wilcoxon signed-rank test for ordinal variables. Medical visits were assessed for the intervals of 6 months before and after intervention. Only diabetes-related complications were considered for the emergency department visits and hospital admissions.

**Table 3:** Variables related to self-efficacy, quality of life, diet, and physical exercise at baseline and 6 months after the intervention.

	*n*	Preintervention	Mean difference (95% CI)	*p* value
*Spanish Diabetes Self-Efficacy Scale*				
Diet	131	6.5 (2.2)	0.5 (0.2, 0.9)	0.006
Physical activity	137	6.7 (2.2)	0.7 (0.3, 1.1)	0.0003
Disease control	136	6.2 (2.1)	0.8 (0.5, 1.2)	<0.0001
Total score	128	6.5 (1.7)	0.6 (0.3, 0.9)	<0.0001
*Quality of life, physical activity*				
ADDQoL score	145	−1.4 (1.4)	−0.02 (−0.2, 0.2)	0.877
Moderate and vigorous activity minutes/week	137	539 (776)	−13 (−144, 117)	0.840
Moderate and vigorous activity MET hours/week	137	5.5 (7.7)	0.01 (−1.4, 1.4)	0.984
Met physical activity recommendations for their age; *n* (%)	137	79 (58)	12 (4, 21)	0.007
*Dietary habits; n (%)*				
Fruit & vegetables: ≥5 pieces p/d	141	37 (26)	10 (2, 18)	0.020
Olive oil: ≥3 soup spoons p/d	142	28 (20)	−4 (−12, 5)	0.398
Red meat: <2 portions p/w	139	47 (34)	3 (−6, 11)	0.505
Cold cured meat: <2 portions p/w	131	64 (49)	11 (1, 20)	0.035
Legumes: ≥2 plates p/w	139	97 (70)	−4 (−13, 4)	0.304
Commercial sweets: <2 pieces p/w	125	87 (70)	3 (−5, 12)	0.465
Beverages: <1 can p/d	124	107 (86)	−2 (−8, 3)	0.405

Data are mean (standard deviation), unless otherwise stated. *n* (%) = frequency (percentage). The “*n*” column reports frequencies of available data at both time points. ADDQol: Audit of Diabetes-Dependent Quality of Life. MET: metabolic equivalent. Meat portions were 100–150 grams for red meat and 4-5 slices or 80 grams for cold cured meat. p/w and p/d indicate per week and per day, respectively. Differences were calculated as postintervention minus preintervention values. Mean difference for categorical variables corresponds to differences in paired proportions and their respective 95% CI. Reported *p* values are based on paired *t*-tests for continuous variables and McNemar's test for binary variables.
