# Supplementary material for: A Pilot Study to Assess the Feasibility of the Spanish Diabetes Self-Management Program in the Basque Country
Source: J Diabetes Res. 2016 Dec 29;2016:9145673. doi: 10.1155/2016/9145673 (PMC5227166; doi:10.1155/2016/9145673)
Supplement: Supplementary file 1 — Satisfaction survey questions. [file 9145673.f1.pdf]

SATISFACTION SURVEY OF ACTIVE PATIENT PROGRAM

The Active Patient Program hopes this course has satisfied your expectations and now you´ll be able to better manage your disease.

We kindly ask you to take a few minutes to answer the following questions. Your opinions are very important to us and will help us improve this course.

Please do not forget to bring this document filled in on the last day of the course.

Thank you for your cooperation.

START DATE COURSE____

PLACE OF COURSE____

PERSONAL INFORMATION

Sex: Male ☐ Female ☐ Age: ___________________________

Have you missed any sessions of the course? If so, what was the reason?

- ☐ illness or fatigue
- ☐ did not have time
- ☐ transportation problems
- ☐ dissatisfaction with the course
- ☐ another reason:

This is a translation of the satisfaction survey used in the pilot study

SATISFACTION SURVEY OF ACTIVE PATIENT PROGRAM

Please express your level of agreement with the following statements about overall satisfaction with the course (1 strongly disagree to 5 total agree):

	1	2	3	4	5
1. The place and schedule of the course seemed appropriate to me					
2. The course instructors explained the course concepts in a didactic and easy to understand way					
3. The instructors were adequately informed and trained					
4. I trust the information and instructions received during the course					
5. The concepts taught during the course are clear, well structured and adapted to our culture					
6. In general, I believe that the course is well organized					
7. I managed to communicate in a natural way with instructors and other participants					
8. In general, I am satisfied with the course					
9. I would like to attend another similar course, like this one					
10. I would recommend this course to friends, family, etc. who are in a similar situation as mine					
11. Thanks to this course, I got to know my disease better					
12. I believe I will improve my relationship with my doctor / nurse					
13. I will improve my relationship with my family and friends					
14. I will improve my diet, considering the particularities of my disease					
15. I will change my physical activity habits					
16. I believe that the course will help me to deal better with pain					
17. The course will help me to better manage difficult emotions, like sadness or anxiety					
18. The course will help me to set and achieve objectives in relation to my disease					
19. My confidence has increased and this will allow me to have better control over my drugs treatment					
20. In general, the course will help me to better manage my disease					

This is a translation of the satisfaction survey used in the pilot study

What are the 3 things that you less liked, or you would have changed in this course?

What are the 3 things you liked most?

Have you got any further comments of or suggestions to make?

Would you be interested to keep in touch with other course participants?

Yes ☐ No ☐

Would you be interested in participating in the program as an instructor of other patients?

Yes ☐ No ☐

If so, please mention it to your instructors or send an e-mail to: pacienteactivo@osakidetza.eus

This is a translation of the satisfaction survey used in the pilot study
